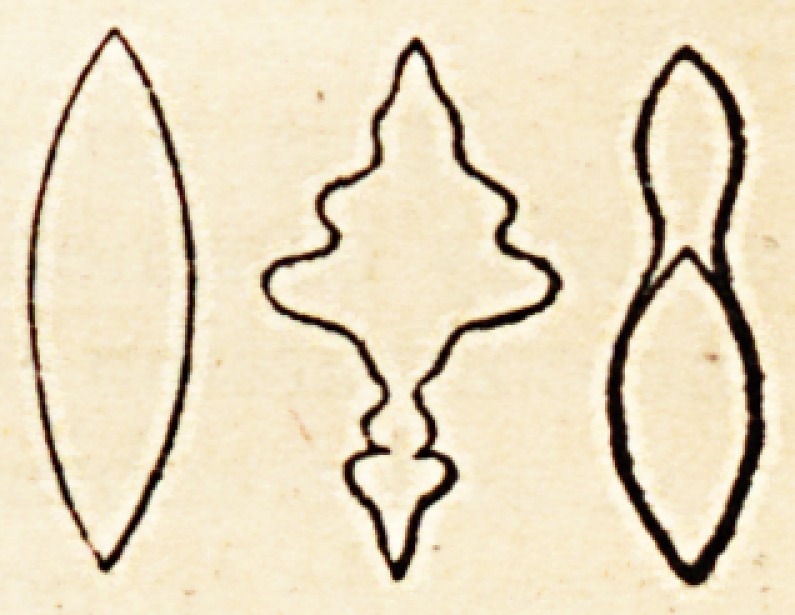# Mental Dynamics, in Relation to the Science of Medicine

**Published:** 1852-10-01

**Authors:** Stanhope Templeman Speer

**Affiliations:** PROFESSOR OF PHYSIOLOGY IN THE UNIVERSITY OF MONTPELLIER; CHELTENHAM


### Original Communications

### MENTAL DYNAMICS, IN RELATION TO THE SCIENCE OF MEDICINE.

A COURSE OF LECTURES DELIVERED BY M. LORDAT, PROFESSOR OF PHYSIOLOGY IN THE UNIVERSITY OF MONTPELLIER. ARRANGED AND TRANSLATED BY STANHOPE TEMPLEMAN SPEER, M.D., CHELTENHAM.

#### Lecture II.

Gentlemen—It is impossible to speak of senescence and insenescence, without at the same time considering that of which they form an integral portion—life. I have already alluded to this in a general way upon the occasion of my opening lecture, but I now shall renew the subject in a more didactic manner; this I trust will be of service to us, not only in our investigation of the chief object of the present course, but also in enabling us to obtain definite notions of life itself; inasmuch as the term by which it is usually expressed, is employed in an acceptation so vague and so *arbitrary*, as to become the cause of numberless controversies. I think that it may, however, be possible to avoid these dissensions, through the medium of an accurate definition.

What then is life, taken in its most general acceptation, and such as we see it in all things possessed of vitality, to whatever kingdom they may chance to pertain?

Linnæus, in his "Philosophia Botanica," thus expresses its elementary constituents, when saying that the reality of life, as it exists in a given body, is proved by the following phenomena:—"Ortus, nutritio, ætas, motus, propulsio, morbus, mors, anatomia, organismus." To define the same object, I shall also endeavour to construct in our own language a sentence combining the majority of the above characteristics, together with a few others which I conceive to be of importance.

Life is a *temporary phenomenon*, consisting in this, that a *uniting* principle, proceeding by *succession* from a living aggregate—primitively infinitesimal, inconceivable, and formative—arranges and *constructs* slowly, from a *variety* of *heterogeneous* and *incompatible* elements, and at the same time *maintains the integrity* of, a combination eminently unstable and perishable; in which, however, it *carries on a plurality* of conservative functions—*expands, developes* itself, acquires its maximum of intensity, and at a given period commences its retrograde course and consequent tendency to extinction; a result which at length takes place, without the primitive aggregate having lost the *conditions essential* to the *habitation of its original principle,* which at its departure leaves its quondam tenement at the mercy of those *destroying agencies* to which it is physically liable by the heterogeneous nature of its elementary composition.

I confess, gentlemen, that this protracted definition has left me almost breathless; but, in spite of the objections that might be urged against it on the score of taste, I shall adopt it, if intelligible to my hearers; and to assist in rendering it so, let me offer a few comments on each word that has been pronounced with an intentional accent.

1st. *Life is a temporary phenomenon.*—It is not the permanently infinite condition of a body. It is not a quality of matter—a property of substance—as are the forms of crystal, or the physical and definite characteristics of any given material. It has rather an approximative duration, the span of which varies in different species; but is nearly constant in the different individuals of the same species. Nor are the successive periods of this interval of time indiscernible; on the contrary, each one may be distinguished from its successor and predecessor, not only by its position, but by forms, functions, and aptitudes peculiar to itself. All the known phenomena of life have been estimated by their period of duration, or, in other words, by their commencement and termination.

2nd. *A uniting principle*—Connecting, unconsciously as it were, its varied operations, whether simultaneous or successive, by a process similar to that by which we perceive certain actions to be in conformity with the preconceived projects of the intellectual principle itself.

3rd. *Proceeding by succession.*—This principle is not of spontaneous creation—in other words, is not of abstract origin; nor, on the other hand, is its formation due to general causes of a physical order. It is begotten, neither by the laws of mechanics, of chemistry, nor yet of any imponderable cause whatsoever; but it proceeds from a living body. We know that it is transmitted from an ascertained source of parentage, or at least from an aggregate, that enjoyed an existence either at or before the period of its birth. We acknowledge no spontaneous generation; and if the progenitors of a living being have not the same form that it itself possesses, the cause is to be sought for in this, that the vital force on the maternal side has possessed the faculty of creating parasites—a power which is found to exist in a variety of species belonging to the different kingdoms of nature (intestinal worms); or else that the elements constituting the aggregate of the maternal progenitor were themselves the product of a parent, similar to the living object at present under our notice (worms produced in the decomposition of animal matter). Harvey has said, that every living being was of ovular origin (and let us hesitate before denying the veracity of so great a man); for it is certain that we see none, that have not their origin at least, in another living being.

4th. *Infinitesimal.*—I thus denominate a principle, which is susceptible of growth, but which at its commencement is possessed of dimensions, to us inappreciable and incomprehensible.

5th. *Unimaginable,* as regards its nature. We can compare it to nothing that comes within the scope of our senses, nor yet to aught that the imagination may create. We know of it but the actual existence and causal power; the remainder is beyond the limits of our finite understanding.

6th. *A plastic or formative power.*—In the production of a living body there exists a cause, which is not the power of crystallization, the power of cohesion, nor any known or unknown chemical or physical agency. It is *plastic;* that is to say, it fashions an aggregate, and models it into shape far better than could a sculptor, inasmuch as, not content with imprinting upon it an external configuration, it gives a form to all its internal parts. The plastic or formative process can have no connexion with that of crystallization, since in this the general appearance is but the result of an accumulation of elementary shapes, all of which are identical . . . . . whilst in the former the elementary principles are amorphous, and each individual form has necessitated for its formation a special act.

7th. *The plastic principle unites and maintains in contact, elements in themselves heterogeneous and incompatible.*—Yes! these elements consist of atoms which no natural affinity could attract, and, as such, the intervention of an unseen influence becomes necessary, to bring them together. I have said, that these heterogeneous elements are of themselves incapable of association (at the risk of being guilty of a pleonasm), only to prevent any misconception relative to the activity of that power which retains them in one definite combination, in spite of affinities recognised by the laws of chemistry. Vainly do these atoms tend to repel each other, to form new compounds, to obey diverting or diverging causes; an irresistible power constrains them to a forced quiescence, and their natural and physical affinities can only be exercised, when this same power shall have been destroyed.

Many of my auditors have doubtless read a witty satire, entitled "Medical Art; or, The True Secret of Success in Medicine." Probably they may have noticed in it a remark directed against the advocates of the polypharmic system, who pride themselves on the multiplicity of drugs they can administer in one prescription, and value these only in proportion to the number of ingredients they contain; although, as in the language of the satire alluded to, these very ingredients "may curse the hand that has brought them together."

Well then, gentlemen, that which ignorance and quackery are daily guilty of, in the money-getting department of our profession, is done by the vital force, in order to form the crasis of its aggregate; or, to repeat the words at the head of this last proposition—*The plastic or uniting principle maintains in contact; elements in themselves heterogeneous and mutually repellent.*

8th. *The creative power of vital phenomena, performs certain functions, increases and develops itself;* attaining finally its maximum of intensity. The series of these functions is the continual manifestation of life, and the means of its conservation; their interruption for a sufficient length of time producing, not only the deterioration and extinction of the vital force, but, in addition, the destruction and consecutive dissolution of the entire system. The functions alluded to, are of two kinds:—1st. Those pertaining solely to the integrity of the aggregate itself, furnishing it with the means of fulfilling its destiny without being prematurely exhausted. 2nd. Those, the effects of which are mainly directed to the welfare of the species, inasmuch as their office is the procreation of an individual, similar to the original.

The functions included under the first head remain in exercise during the whole of life; their activity being proportionate to the intensity of the vital force, considered in relation to the epoch of its duration. Those pertaining to the second series—viz., the generative functions, are limited to a certain period of life.

The manifestation of these functions varies in the different species. In some, that of generation begins at the time that the system has attained its full maturity. It is thus with the silk-worm. In the salmon, the generative power is evinced by the female, only on attaining the adult condition; whilst in the male it comes into play, when but a few inches in length.

Diseases, again, are the expression of various fluctuating conditions of the vital force. In one point of view, indeed, they may be regarded as functions, since they consist of acts which tend to a useful purpose, but in the course of which the said power may fail.

In the development of a living aggregate, two points require to be considered separately—the process of growth and that of invigoration. It is easy to perceive that they are distinct, although generally running parallel the one with the other. The vigour of the system may be susceptible of daily variations; the dimensions of the body, however, are incapable of experiencing such sudden changes. In considering, however, the aggregate from a more general point of view, we find the principles of growth and invigoration to be so interwoven, even from the origin up to the maximum of intensity of the vital force, that if the well-being of the system be in any way interrupted, it becomes often a matter of difficulty to determine which of the two has taken the initiative. Has a physical impediment to the process of growth produced the diminution of strength? Or, has the diminution of strength rendered the growth tardy? Let it, however, be remembered, that after the culminating period has passed, the above relationship no longer exists.

9th. *After acquiring a maximum of intensity, it begins its normal downward progression.*—The maximum of development in a living aggregate, occurs at that particular point of time, which divides the natural span of life into two distinct and equal periods; the one of augmentation, the other of declension. Its corporeal value having increased at first both in dimension and aptitude, then gradually experiences a diminution, not indeed of dimension, but of functional capability.

In each species there exists a definite proportion of time between the duration of development and that of degradation. In some, this proportion is about equal; in others, the period of development is long as compared with that of decay. Let us take, for instance, the silk-worm. Its transformation into the butterfly must be considered as its period of apogee; but we know that this brilliant appearance is but a speedy forerunner of death. So it is with many annual plants. Behold the contrast between their vital existence and that of shrubs and trees.

If, however, after that the culminating point in the career of the vital principle has been attained, there be not invariably a diminution in the weight and volume of the aggregate material; and if, as is even possible, there should happen to be rather an augmentation of these properties, there is at least, and without exception, a withering which constitutes an indisputable sign of antiquity. As being the result of debility on the part of the vital principle, it may indeed deceive us by manifesting itself at an earlier or later period than usual, but the error can be but of short duration.

10th, and lastly. *Disappearance of the vital principle before the aggregate has become sensibly uninhabitable—necroptic decomposition.* The reduction of this aggregate to a cadaveric condition, must of necessity enter into the definition of life as an essential characteristic. Those who maintain that life is but the result of an instrumentality on the part of the aggregate, overlook this fact. They would wish us to believe that the cessation of life is the effect of mechanical deterioration and exhaustion. But this assertion is either an error or a falsehood; and we know full well those cases in which the decline of the vital principle has been accelerated, by some alteration capable of marring its conservative functions. The truly scientific physician may well deserve our confidence when he affirms, "That in the great majority of deaths, whether senile or premature, the anatomico-pathological appearances are insufficient explanations of the same."

Here, then, we have a series of ideas united in such a manner as to afford a general notion of life in beings of every description, from the lowly moss to the cedar of Lebanon,—from the lowest form of infusoria up to man himself. I affirm, that a body is endowed with vitality when I see that it is the seat of those transitory phenomena which I have just described.

Certain materialists, again, would have us believe, that the gyratory movements of particles of camphor on the surface of water, and also those produced during the formation of some chemical combination noticed by M. Geoffroy-St.-Hilaire, should be looked upon as "the rudiments of life." But I would ask, what relation do these said movements bear, to the series of elementary phenomena which I have just described as in truth constituting life.

Cabanis, unwilling to allow that it can be derived from any other source but that of physical phenomena, says—"The conditions necessary to the manifestation of life in animals are not, probably, more beyond the reach of discovery than those from which result the composition and formation of water, hail, and snow; or the production of many chemical compounds, possessed of properties entirely different from those of the elementary principles from which they have been formed."

The question, however, is not to ascertain whether it be more difficult to discover the theory of life than that of the composition of water, hail, &c. What it imports us to determine is, whether from the form, succession, and co-ordination of certain appreciable facts, phenomena of a vital and physical order can be attributed *à priori* to a set of causes alike unknown to us. The transitory nature of the phenomena of life, its hidden powers, its faculty of uniting molecules, otherwise insociable, its progressive ascension, culmination, and declension, its annihilation, without obvious or sufficient physical cause, a series of conservative functions . . . . do these facts belong to the same order of causes that give rise to the storm and thunder of summer, the snow and ice of winter? An answer is necessary if we aspire to the creation of a science. Common sense suggests to us the propriety of suspecting hidden agencies, by a consideration of the relationships and diversities, which we see to exist in their effects. Hence it is that philosophy has instituted two distinct series of causes, the one of physical, the other of metaphysical origin. But Cabanis, an ardent materialist, admits no such distinction. With him all is the result of blind necessity; we must not employ such terms as *why*, *wherefore*, *design*. Doubtless you have hitherto been credulous enough to suppose, that eyes were created for the purpose of seeing, teeth for the mastication of food. It is absurd, however; these facts are as much the result of blind necessity as the fall of an antique tottering building. But I ask you, is this science?

Some of the ancient writers have discussed at length what they term the vitality of the world. Lucretius describes the phases of its existence, and even its old age. This, however, is but the offspring of poetic licence. Does it become you or me to speak of the life of an object of which we know neither the origin, growth, development, decay, dissolution, or decomposition?

What, then, shall we say of the dogma of Strabo, Spinoza, and Campanella, that everything is endowed with life? How is it possible to apply such a term to this marble table, to this pulpit in which I stand, to those benches on which you sit? I see in them none of that succession of phenomena which, in my opinion, constitute life. Let others assert, if they will, that there exists everywhere an activity, a tendency to motion; well and good; but life is something more than mere motion.

Among the numerous theories of Spinoza regarding the life of man, there is one, which some have considered as ingenious, others, as far-fetched. . . . It is this—viz., that the vitality of the living human aggregate is but the material aggregate itself, seen from one particular point of view; and that in man as a living being, there has been no diversity of causes at work.

Now, common sense tells us nothing of the kind. On the contrary, it teaches that there really exist three causes, which it is impossible to include in one category: 1st. The cause which brings together and retains in contact the molecules. 2ndly. The molecules themselves. 3rdly. The principle of intelligence. Neither of them is of necessity derived from the other, and they are adventitious, each in regard to the other. The vital principle is anterior to the formation of the material aggregate, and the principle of intelligence depends neither upon the vital principle, nor upon the aggregate material, since a child without brain or spinal cord may live for a certain time, though utterly devoid of the principle of intelligence. A recent corpse can engender neither this principle nor that of vitality. It becomes, therefore, essential to consider a diversity of causes separately, and not one self-acting agent, regarded under as many varied aspects as we may choose. If the assertion of Spinoza be not a riddle, it is either a mystification or an absurdity.

But I am wrong in thus speaking; it is rather an unintelligible artificial language, instituted merely to accustom the mind to his fundamental doctrine; the unity of matter. When we wish to affirm that God is not distinct from the world, it may reconcile the hearer to so startling an assertion if we first lead him to regard any substance whatever, as matter, spirit, deity. But again let me ask, can Science lend herself to so revolting a fiction?

Enough, then, as regards life in general; but as you have been told previously, that the form of its duration differs in different species, which it thus characterizes and specifies, it becomes necessary to take a bird's-eye view of the circumstances which modify the varied features of this abstract sketch, in order to apply it to man.

We shall not dwell long upon the early periods of life. Pliny has said, that man at the moment of birth is the most miserable of all animals; that which most needs the assistance of his fellows; and we must allow it. Man is born with no other instinct than that of breathing, crying, and swallowing. How far removed is he from the foal that runs as soon as born; the chicken, that seeks its food as soon as it emerges from the shell; or the nest bird, which, perceiving the approach of its parents, raises its head, extends its neck, opens its beak, chirps, &c.

We confess, therefore, that at the moment of birth, man, whose uterine existence constitutes about the hundredth part of his natural life, is, of all newly-created beings in the animal kingdom, the least advanced in biologic development.

Were we to apply to a man at birth, the term elephant, buzzard, goose, mule, &c., we should certainly pay him a compliment; as, however, after the lapse of twenty years, its repetition would undoubtedly be received with very bad grace, it stands to reason, that in the interval he must have indemnified himself to no small extent. What, then, are the advantages he has acquired?

Man has undergone a process of development (similar to that of animals) in proportion to the duration of time peculiar to the life of his species. As in brutes, so in him; all those functions pertaining to his preservation and propagation have been active. He has therefore been their equal in this respect. But what has placed him before and above all, has been the expansion of a hitherto latent principle, constituting in him a being beyond the pale of all other living objects.

Thus, we find in the life of man two parts which it is impossible to regard in the same light, but which require to be studied separately and in detail. The first is that, which emanates from the vital plastic principle, and is similar to that of all other living beings, and especially of animals. The second is the intellect itself, the activity of which is only manifest after birth, and cannot possibly be placed in juxtaposition with the life of these same animals. The former consists of everything relating to the formation and maintenance of the material aggregate, or to the furtherance of the species; the latter is the representation, as it were, of an epic poem, the varied subjects of which are for the most part independent of any interest in the stage on which they are represented. And thus the life of man affords us two distinct subjects of investigation—first, the aggregate and its preservation, which we designate, the canvas or rough draught of the individual . . . . and secondly, the series of scenes represented upon it, which are the work of the intellectual principle. It is the comparison of these two objects which at present occupies our attention. Let us therefore first examine the proceedings of the formative or plastic process. To this I shall apply the term zoonomic life, or that which is conformable to the laws of the vital constitution of animals; reserving the term, intellectual life, to that portion of human existence controlled solely by the principle of intelligence itself.

Let it not however be supposed, that all the functions essential to the preservation of the individual and his species, are uninfluenced by this latter principle. I am well aware, that in man, when instinct is limited, the more important functions of relation require the co-operation of the intelligence.

I do not, however hesitate, to include under the head of zoonomic life, in man, every function analogous to what takes place in certain animals living in a state of nature. But I must be permitted also to comprehend under the head of intellectual existence, those additional functions of which animals are incapable.

This distinction having been made, it becomes essential to compare the intensity and progression of these two forms of elementary life. I must, however, explain the meaning I would here attach to the two words, *intensity* and *progression*,—I have previously had occasion to institute a comparison between the vital force and the intellectual principle, in relation to their individual aptitudes; such comparison then has been *qualitative.*

But these two principles may be compared in a mathematical point of view; in relation, for example, to the amount of activity, and to the rapidity of successive acts, &c.; constituting their *quantitative* value.

Now the points of comparison which I purpose to institute, between the zoonomic life and that of the intelligence in man, while seeking to ascertain whether they alike undergo such changes as youth, culmination, senescence; belong to this latter category—*i. e.*, they are quantitative. I include them in the terms intensity and progression. The former expresses the amount of functional activity, regularity, tenacity, and endurance. The latter constitutes the order of succession in which augmentations and diminutions of the vital force take place.

Having advanced thus far, let us inquire as to what are the most certain facts connected with the intensity and progression of these two divisions of human life. We may commence with that which I have styled the Zoonomic.

1st. The Vital Principle, possessed of so little tenacity, so little power of endurance, as to be annihilated with the greatest facility, possesses, nevertheless, a prodigious activity. In the space of nine months, or less than the hundredth part of a natural life, it has succeeded in forming a perfect system, the further increase of which takes place more slowly after this period.

2nd. The development of the aggregate, both in dimensions and in aptitude, continues up to about the middle period of life—that is, to forty or forty-five years. It may take place at one time in height, at another in consistence, at another in weight, but always in vigour. Formerly it was supposed that a real vital increase took place up to this period; it was, however, but a conjecture. Now, however, and since the laborious investigations of De Parcieux on the mortality of the human race at different periods of existence, it has been shown by Barthez, that towards the middle period of the normal duration of life, there is in reality a true increase of vital capacity and aptitude, evinced by its augmented powers of endurance and tenacity.

3rd. After this epoch there ensues a declension, the course of which is analogous to the previous progressive ascension. It has been remarked that there not unfrequently occur irregularities, which mar the otherwise continuous course of progression and retrogression.

4th. Cases of longevity usually present an equality in this particular; if retrogression be slow, progression has been so likewise.

5th. True senile death, consists in a simple extinction of life without disease; such as that of Fontanelle, who merely felt, when at his last gasp, the difficulty of continuing to exist. This termination, however, is rare; more generally it is accelerated in a greater or less degree by some disease, which, however trifling, proves sufficient to occasion a premature and hurried retrogression.

6th. At any period during the course of life, its phenomena may be suppressed, whether by some violent disorganization of the system, by the suspension of one of those functions denominated vital by Galen, or by an accidental encounter with certain destructive influences, as of poisons, or of deleterious miasmata, &c. This sudden termination of existence is too common to need particular notice.

7th. One thing, however, I must press upon your attention, which is, that at any period of its course the vital principle may undergo a fatal retrocession, tending, indeed, to abridge the natural duration of life, but not to be regarded or confounded with violent or sudden death. The system at the time may be in full vigour; but from some constitutional peculiarity, or from the supervention of some malady, a premature retrogression of the vital powers takes place and proceeds with more or less rapidity. The ordinary functions become feeble, imperfect, at length cease, and the vital force itself is prostrated and finally extinguished.

This irrevocable declension on the part of the vital principle may always be looked upon as an old age more or less *accelerated*, whether it occur by *anticipation*, or at the *legitimate* epoch. Thus the majority of acute and chronic diseases terminating fatally, but lasting a considerable time without rendering the vital organs *utterly* inadequate to perform their necessary functions, are cases of *accelerated* senescence.

As most of these facts may be considered as quantitative comparisons, it is not very difficult to express them somewhat geometrically, by lines and figures. You are aware that algebraic and chemical truths have been thus usefully demonstrated. Let me, therefore, employ the same means, to fix in your memory the physiological truths which I wish to establish.

The figures I propose to employ, are imaginary solids. I show you, however, merely their outline.

The temporary duration of life may be represented by the figure of a spindle, one point of which stands for the first moment of existence; its gradual expansion corresponding to the periods that succeed, up to the full development and culminating point of the vital force; while the gradual tapering of the spindle, from its centre to the opposite extremity, represents truthfully the different phases of old age, and its termination in a point, similar to that which served to mark its commencement.

To render this simile more exact, it is better not to employ the mathematical spindle, thus—

composed of two pyramids united at their respective bases, the outline of which would represent a rhomb.

In such a figure the culminating period would be indicated by a mere line. But this same period is not thus indivisible; it is, on the contrary, of some duration, and may leave us for some time uncertain as to whether the vital principle is approaching or departing from its meridian. To imitate this uncertainty in a figurative point of view, it were preferable to employ the outline of an ordinary spindle, made so that sections of the central part shall afford a number of circles, scarcely larger the one than the other. This figure, then, bear in mind, not only affords a type of the duration and progressive tendency of life in a zoonomic point of view, but accurately corresponds in its central part, to that somewhat uncertain period at which culmination takes place.

Another remarkable circumstance connected with this phase of existence might be graphically represented. I allude to the variations which take place in the vigour of the creative powers, without altering the general form of the collective phenomenon. At all ages diseases may occur, and during their manifestation there is not unfrequently a diminution in the intensity of the vital principle; but when such diseases have terminated, then follows a period of convalescence, and often, of increased health and vigour. As a rule, however, these alternations have but little effect on the general course of existence; the retardations which it may experience in youth, do not prevent an onward progression up to a stated period, while the reinforcements it may perchance receive during its declension are insufficient to obviate the tendency to final extinction.

The expression of the above fact may be figuratively represented by the varied ornaments carved upon a spindle by means of a turning lathe. However profusely decorated, you may always recognise the two extremities and the central expansion, and if the plan of such a body be represented on paper, the outline will be more or less scolloped; but the general form of the polygon will always be that of a curvilinear rhomb; thus,

With regard to the premature senescence mentioned by Galen, nothing can be more easy than to represent it. Draw a diangle, thus—which in its general outline, A B C D, shall represent the normal zoonomic life, and note especially the period of culmination, B D. From the point A, draw two curved lines up to the point at which the vital principle begins to decline, B D, and continue them up to the point C, representing the period of extinction. In the area of the polygon draw lines indicating the moment at which the vital principle deviated from its natural course, and unite them at the points E F.

Below the line which represents the culminating period B D, you perceive parallel lines G H I K indicating the individual culmination of premature senescence in different instances. Under this head you may reckon those who have died before the age of forty, not from any violent cause, but from some disease which the vital principle has been unable to overcome. Such were the learned Pic de la Mirandole, to whom a passionate love of study proved fatal; the gentle Raphael, whose devotion to the art of painting shortened his existence; the wise and precocious Vauvenargues, prematurely hurried to the grave at the age of thirty-two; and the delightful Mozart, whose untimely end at the early age of thirty-six might have been easily predicted.

The transverse lines above that of the normal culminating period, will call to mind those men, who, having attained this period, arrived at the close of their existence in a disproportionately short space of time; in other words, the second half of their vital career underwent curtailment. Such were Bacon, Descartes, Racine, Barthez, De Candolle, and many others, whose primitive constitution promised an equal duration of existence on either side of the culminating point, but of which the declining period was unexpectedly shortened. Here, then, we have three forms of which the zoonomic life is susceptible; one representing its natural course; the second, the variations which man often undergoes in regard to health, and in which certain compensations take place, permitting the individual (in spite of suffering and danger) to arrive at the natural term of life; and the third, in which the early period of life follows a natural course up to a certain epoch, while the remainder undergoes a declension so rapid and sudden, as to bring its terminal point on a level with the normal period of culmination. The first of these figures being that of an ordinary spindle; the second, that of a carved or scolloped spindle; the third, that of a spindle with a head; thus—

I purpose now, to submit the intellectual career of man to a similar geometric configuration, in order to ascertain what may be the outline of such an imaginary solid, as compared with those which I have just described.

In doing this, I shall confine myself to the same points which have been considered in relation to animal existence, namely, the *intensity* of action and its *progressive* career. I embrace the entire range of the intellectual principle, just as in the case of the vital force, including the respective attributes of both, and shall proceed to institute a comparison between them. I may eventually be obliged to request you to omit one of the intellectual functions, the operations of which take place frequently with the co-operation of the vital principle: I allude to the memory. During the past year I insisted strongly on the part which the vital force performs in the exercise of this faculty. You will remember how I showed that imperfections occurring in the operations of the memory, by no means imply an enfeebled condition of the intellect.

Previous to examining the career of the intellectual principle after the culminating period of the vital force, it may be useful to compare the relationship which exists between the two, during the first half of existence.

1. The first point to be noticed, is the fact that the initiatory date of intellectual capacity is not the same as that of the vital principle. This last commences its operations immediately after conception, without losing any time; every minute is registered, inasmuch as if the birth be premature; the fœtus gives well-marked indication of how much was wanting to complete the full term.

It is not thus, however, with the principle of intelligence. Its activity would appear to commence but at the moment of birth. Not that there is any reason to believe that the formation of the human dynamism has been instituted at two distinct periods. The formative or vital principle, together with that of the intellect, must have started into being, simultaneously; but while the former has at once entered upon a career of activity, the latter has remained in abeyance, until the period at which the aggregate should manifest itself to the external world. We might be led to imagine that the intellectual principle remains latent and inactive, simply from not being liable to the impression of objects, capable of eliciting sensations and affording it an opportunity for the formation of ideas. Experience, however, proves that this is not the case; and that by a primordial disposition, the principle of intelligence remains as it were in seclusion, until the natural term of utero-gestation be accomplished. Should an unforeseen accident induce the premature expulsion of a viable infant, such precocious birth will profit the intellectual principle but little. The child exists much as it has already done during the seven or eight previous months in its mother's womb, unconscious of the external world, except as regards the air it breathes—lulled into a species of continued sleep—generally motionless, or moving its limbs merely by instinct. In the *Gazette Médicale de Paris*, there appeared lately an account of an infant that for the space of six weeks led an intra-uterine mode of existence, while in its swaddling-clothes and cradle. The dawn of the intellectual principle becoming manifest only at the period when natural delivery should have taken place.

In reducing, then, the modus operandi of the human intellectual principle to a figurative representation, it should be borne in mind, that it is not contemporaneous with that of the vital force, and that its activity begins at a later period; while its original cause has nevertheless remained in abeyance from the first.

2. It is no easy point to determine the moment at which intellectual activity commences. Its date of birth would appear to be simultaneous with the conversion of sensations into ideas, or with the first evidences of co-ordination and combination in such ideas. Even in a practical point of view, it is impossible to point out the first act of the will, and consequently the first consciousness of intellectual motive, inasmuch as the effects of instinct are for a long time confounded with those of reason. What a contrast, then, between the primary acts of the intelligence and those of the vital principle; the former, as it were, ignorant at birth, feels its own way slowly and uncertainly. The latter requires no such apprenticeship; its first efforts are masterpieces.

3. The intellectual principle having once conceived an idea and exercised the power of thought, becomes gradually stronger and stronger. Thus the vital principle and that of the intellect mutually strengthen one another—as Lucretius says—

> " . . . Ubi robustis adolevit viribus ætas
> Consilium quoque majus, et auctior est animi vis."

"Age, in strengthening the limbs, ripens the intellect, and augments the vigour of the mind." But Lucretius here tells us only half the truth, and he has good reason for concealing the remainder. He leaves us to imagine that the two principles increase in like proportion, and this is not the case. For it so happens, that in conformity with certain primitive peculiarities, and according to different circumstances, the mode of progression varies in different individuals. If there be some in whom the two divisions of the human dynamism are alike perfect, there are far more, in whom one or other principle dominates and flourishes at the expense of its coadjutor.

## Figures and Tables

**Figure f1:**
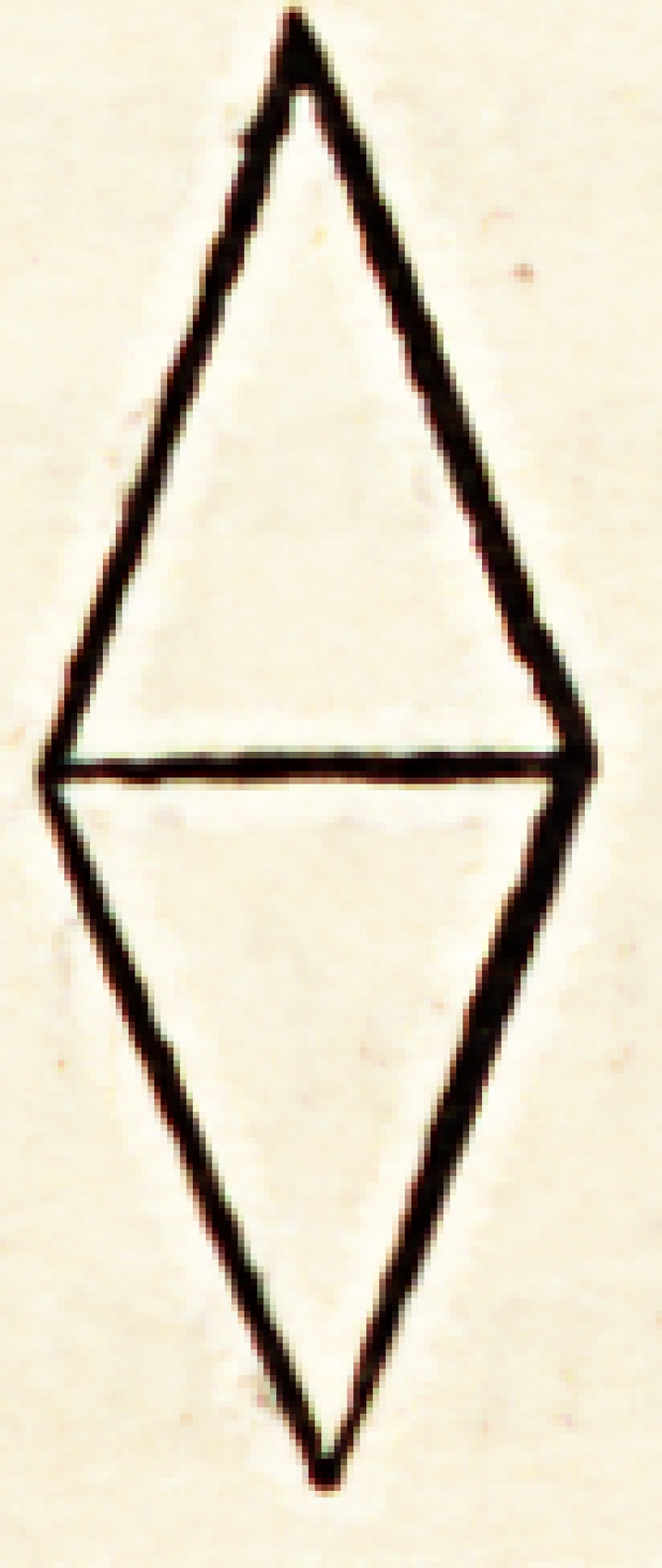


**Figure f2:**
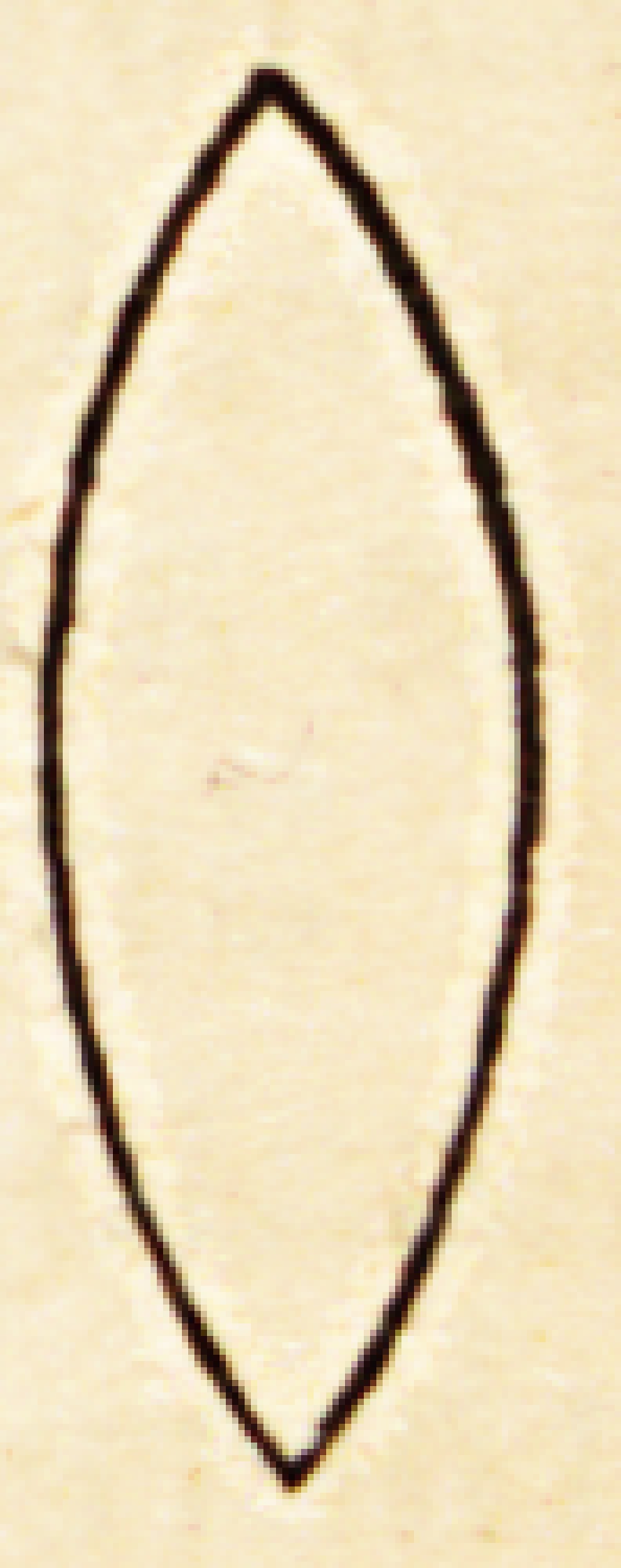


**Figure f3:**
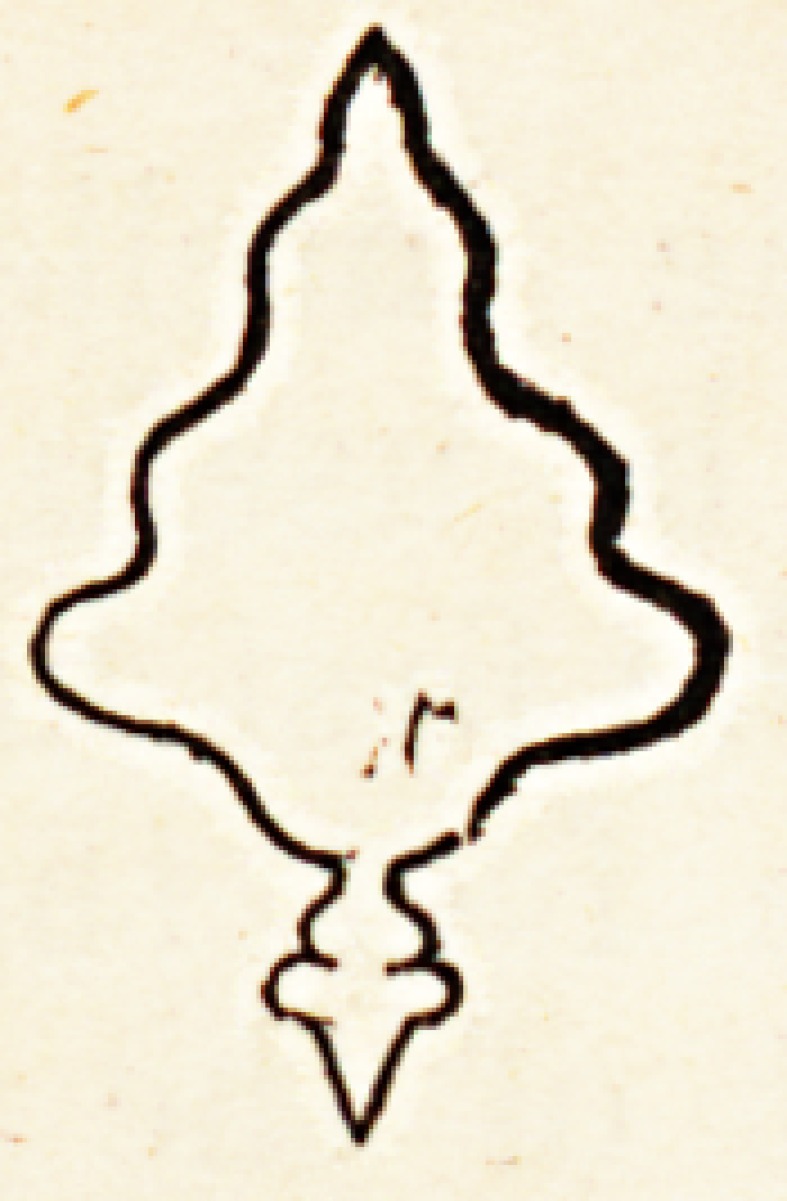


**Figure f4:**
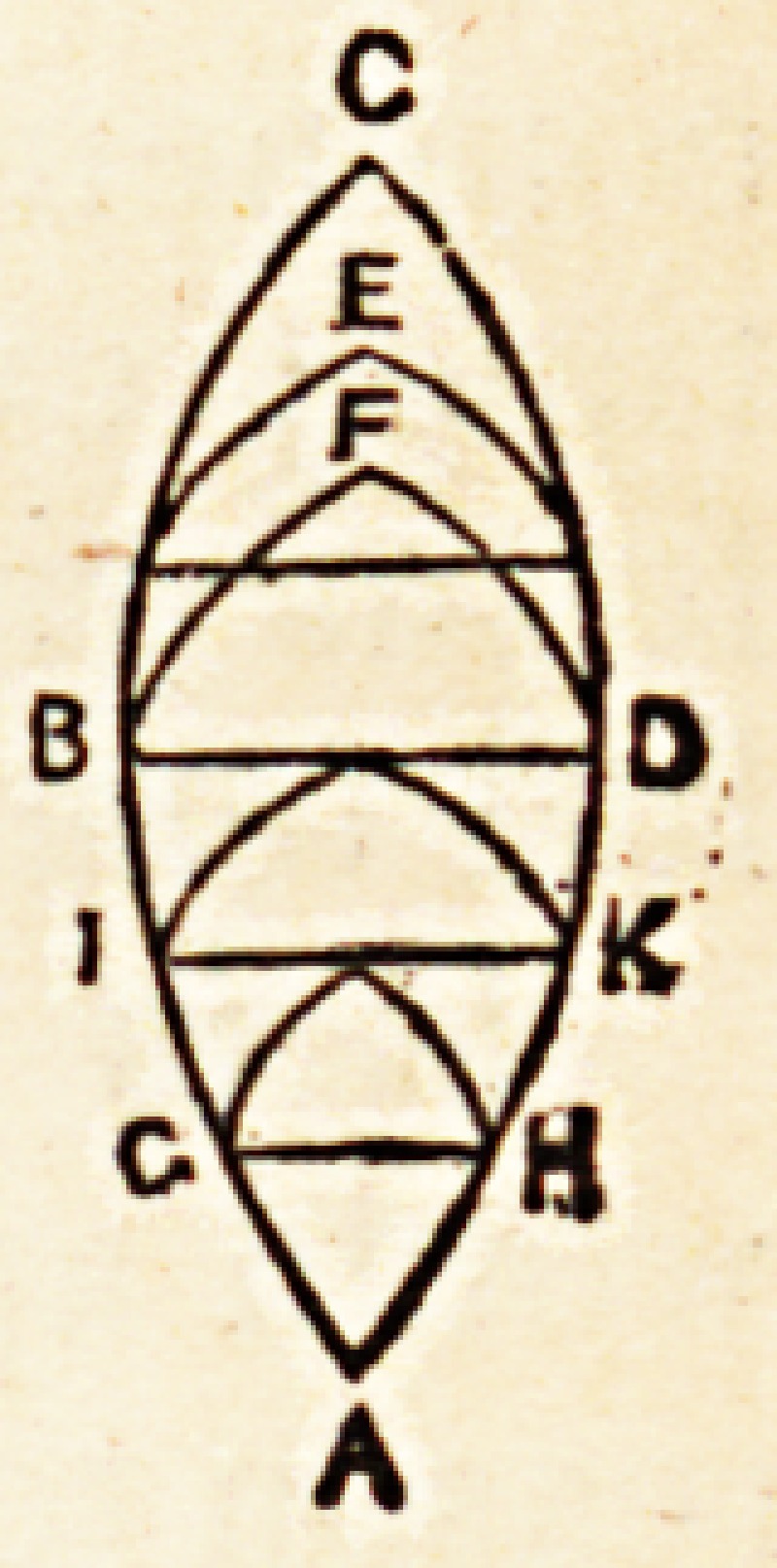


**Figure f5:**